# Gut microbiota modulation as a possible mediating mechanism for fasting-induced alleviation of metabolic complications: a systematic review

**DOI:** 10.1186/s12986-021-00635-3

**Published:** 2021-12-14

**Authors:** Pooneh Angoorani, Hanieh-Sadat Ejtahed, Shirin Hasani-Ranjbar, Seyed Davar Siadat, Ahmad Reza Soroush, Bagher Larijani

**Affiliations:** 1 Obesity and Eating Habits Research Center, Endocrinology and Metabolism Clinical Sciences Institute, Tehran University of Medical Sciences, Tehran, Iran; 2 Endocrinology and Metabolism Research Center, Endocrinology and Metabolism Clinical Sciences Institute, Tehran University of Medical Sciences, North Kargar Ave, 1411413137 Tehran, Iran; 3 Department of Mycobacteriology and Pulmonary Research, Microbiology Research Center, Pasteur Institute of Iran, Tehran, Iran

**Keywords:** Gut microbiota, Microbiome, Intermittent fasting, Ramadan fasting, Metabolism

## Abstract

**Background:**

Intermittent fasting has been reported to have positive effects on obesity, diabetes, cardiovascular diseases, hypertension, and several neurodegenerative diseases through different mechanisms such as alteration in the gut microbiota. This systematic review was conducted with the aim of providing an overview of the existing animal and human literature regarding the gut microbiota alterations in various fasting regimens.

**Method:**

A systematic literature search was conducted on PubMed, Scopus and Web of Science databases up to May 2021 to find all relevant studies examining the gut microbiota alteration during the fasting. Original researches on animal models or human patients were included in this study.

**Results:**

The search fulfilled 3072 documents from which 31 studies (20 animal and 11 human studies) were included. Upon fasting, abundance of several beneficial bacteria including *Lactobacillus* and *Bifidobacterium* shifted significantly. Moreover, some taxa, including *Odoribacter* which negatively associated with blood pressure bloomed during fasting. Ramadan fasting, as a kind of intermittent fasting, improves health parameters through positive changes in gut microbiota including upregulation of *A. muciniphila, B. fragilis, Bacteroides* and butyric acid–producing *Lachnospiraceae*.

**Conclusion:**

The findings suggest that different fasting regimens including alternate-day fasting, calorie- and time-restricted fasting programs and Ramadan fasting could promote health maybe through the modulation of gut microbiome. However, further studies are needed to explore properly the connection between gut microbiota and meal frequency and timing.

## Introduction

Gut microbiota plays key roles in the host’s immunological, nutritional and metabolic functions such as sustaining gut homeostasis for host, fermentation of indigestible dietary fibers and production of essential amino acids and vitamins [1]. Many environmental and genetic factors have an influence on gut microbiota. Dysbiotic state of gut microbiota has been reported in different health disorders including obesity, diabetes, cancer and metabolic syndrome. However, some interventions including diets, eating habits or supplements could restore or promote healthy microbiota and can be considered as one of the promising approaches for the prevention and treatment of these growing global health problems [2–4]. Increasing evidences indicate that intermittent or periodic fasting provides various favorable health benefits and positive effects on obesity, diabetes, cardiovascular diseases, hypertension, and several neurodegenerative diseases [5–8]. Intermittent fasting is a periodic dietary restriction, which can be performed in different ways including complete alternate-day fasting, modified fasting regimens, time-restricted feeding, Ramadan and other religious fasting [9]. It was shown that intermittent fasting induces its beneficial effects through different mechanisms. It might be a successful intervention to prevent and manage obesity, metabolic syndrome, and its complications due to neurohormonal adaptations [10]. Animal studies show that fasting diets may increase mRNA expression of hepatic clock genes corresponded to favorably alterations of key metabolic regulator enzymes of glucose and fatty acid metabolism expression [11]]. Moreover, fasting diets reduce meal frequency, decrease food stimuli and hunger-related hormones, and ultimately prevent weight gain and its metabolic consequences [12]. Recent studies suggest that the effect of fasting and feeding patterns on metabolism can be closely associated with alterations in the gut microbiota. Change in gut microbiota composition due to fasting has been shown to increase the energy expenditure by converting white adipose tissue to brown adipose tissue. Intermittent fasting also promote the microbial fermentation which in turn lead to formation of some bio products that have beneficial effects on metabolic disorders such as obesity, insulin resistance and hepatic steatosis [13]. Recent evidences have been published in favor of the gut microbial shifts and functional consequences for the host in intermittent fasting regimes [14–16]. However, there is no previous systematic review summarizing and comparing the alterations of the intestinal bacterial composition and functions following the different kind of fasting. In this systematic review, we provide a comprehensive overview of the existing animal and human documents regarding the gut microbiota alterations in various fasting conditions and probable mediating mechanisms in improving overall metabolic health.

## Methods

A systematic literature search was conducted on PubMed, Web of Science, and Scopus databases. All related articles published up to May 2021 were considered for inclusion. Search queries were as following: (“gut microbiota” OR “intestinal microbiota” OR “faecal microbiota” OR “gut microbiome” OR “intestinal microbiome” OR “faecal microbiome” OR “gut microbial profile” OR “faecal microbial profile” OR “gut flora” OR “intestinal flora” OR “intestinal microbial profile” OR “gut microbial composition” OR “faecal microbial composition” OR “intestinal microbial composition”) AND (fasting OR “intermittent fasting” OR “Ramadan fasting” OR “Islamic fasting”). Moreover, other relevant references of articles were also reviewed.

Two researchers independently screened titles, abstracts, and then full-text articles. Disagreements between the two researchers were resolved by discussion and consensus. Studies were excluded if the main text was not available or was not in the English language. Reviews, protocols, conference papers and case reports were also excluded. Therefore, only original researches with original data on animal models or human patients exploring any kind of fasting regimes on gut microbiota were included in the present study. The full text of the papers was reviewed to retrieve the relevant information. The following information about each of the studies was recorded: name of the first author, year of publication, study design, study sample characteristics, microbiota analyzing method and main findings.

In order to evaluating the gut microbiota alterations in various fasting conditions and probable mechanisms in improving overall metabolic health, types of fasting regimens were classified into two main subgroups: 1) Time restricted fasting including Ramadan fasting and 8/16 h fasting program. 2) Calorie restricted fasting including alternate day fasting, water only fasting and the Buchinger program.

Jadad scale was used to assess the quality of included clinical trials [17]. The quality assessment was done by two separate investigators following by consensus-based discussion in case of disagreements. The Jadad score ranges from 1 to 5 and the achieved scores ≥ 3, and < 3 points were defined as good and poor quality, respectively.

## Results

**Fig. 1:**
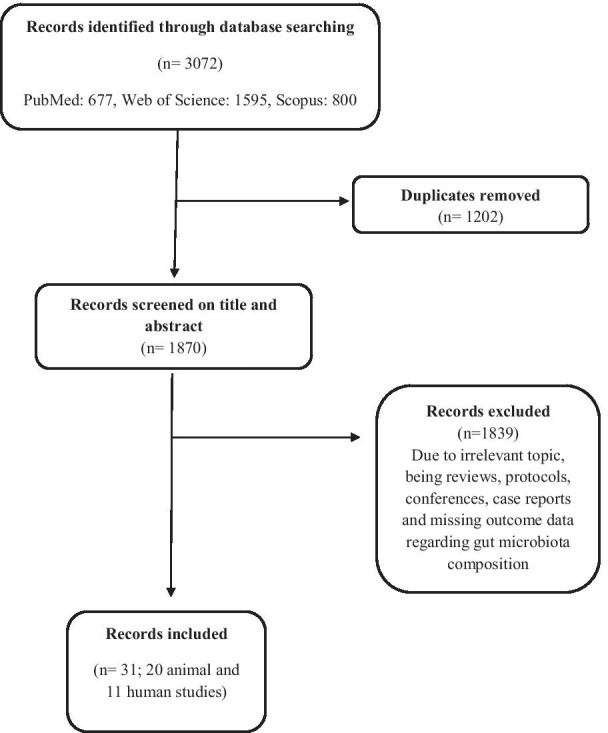
Flow diagram of studies identified

The search fulfilled 3072 studies (PubMed: 677, Scopus: 800 and Web of Science: 1595) of which 1202 were duplicated. 1839 studies were excluded because of irrelevant topic, being reviews, protocols, conferences, case reports and missing outcome data regarding gut microbiota composition (Fig. 1). Finally, 31 studies (20 animal and 11 human studies) were included in this systematic review. (Tables 1, 2).

**Table 1:** Summary of the animal studies investigating the effects of fasting on gut microbiota alterations

First author, year	Animal model	Intervention	Biospecimen	Microbiota analyzing methods	Main findings
Sonoyama K, 2009	Male Syrian hamsters (age: 10 weeks)	1. Fed active group (n = 6)	Cecum	16S rRNA clone library and species-specific real-time quantitative PCR	↑Akkermansia muciniphila, a mucin degrader, in fasting group but not in hibernation
		2. Fasted active, group (n = 6) fasted 96 h			↑Clostridia in fed active and hibernating group
		3. hibernating group (n = 6) maintaining darkness at 4 °C			
Kohl KD, 2014	Fish, toads, geckos, quail, mice	Four time points through	Colon & cecum	16S rRNA gene sequencing	In tilapia, quail, and mice: ↓Lactobacillus & Prevotella, ↑ Oscillospira
		28 days fasting			In tetrapods ↓ Coprobacillus & Ruminococcus
Li G, 2017	7–8-week-old male C57BL/6 N mice: diet-induced obese model	1. Controls: fed with ad libitum (AL) diet	Cecum	16S rRNA gene amplicon sequencing	↑Firmicutes, ↓ Bacteriodetes and Actinobacteria, ↑ Firmicutes to Bacteroidetes ratio
		2. Intermittent fasting (IF): every other day fasting (EODF) regimen			↑ beiging of white adipose tissue and subsequently ameliorate metabolic disorders
McCue MD, 2017	Mice, quail, tilapia, toad, geckos	Mice (3 days fasting), quail (7 days fasting), tilapia & toad (21 days fasting), geckos (28daysfasting)	Feces	16S rRNA sequencing	Alteration in Bacteriodetes, Firmicutes, Proteobacteria, Fusobacteria and Verrucomicrobia
Beli E, 2018	4-month-old db/m (non- diabetic) and db/db (diabetic) mice	1. Controls: fed with ad libitum (AL) diet	Feces	16S rRNA sequencing with the MiSeq platform	↑ Firmicutes, ↓ Bacteroidetes and Verrucomicrobia, ↑gut mucin, goblet cell number, villi length
		2. IF: fasted on an every other 24-h interval for 7 months			↓ plasma peptidoglycan, ↑ tauroursodeoxycholate bile acid ↓retinal TNF-α mRNA
Catterson JH, 2018	fruit flies (Drosophila melanogaster)	1. Controls: fed with ad libitum	Blood and tissue	qPCR quantification of bacterial load	↓age-related pathologies,↑ gut barrier function & gut health due to ↓relative bacterial abundance
		2. IF: 2-day fed,5-day fasted for 40 days			
Cignarella F, 2018	7- week- old Female C57BL/6 J mice: multiple sclerosis (MS) animal model	1. Controls: fed with ad libitum (AL) diet (n = 10)	Feces	16S rRNA gene sequencing	↑*Lactobacillaceae*, *Bacterioidaceae* and *Prevotellaceae* families
		2. IF: fasted, fed every other day (n = 10)			↑ketone formation and glutathione metabolism, ↑ anti-oxidative pathways, ↓IL-17 producing T cells & ↑the number of regulatory T cells ↓inflammation, demyelination and axonal damage
Wei S, 2018	6-week-old male C57BL/ksJ-db (db/db) mice: a genetic model of type 2 diabetes	1. Controls: standard chow, free access to food and water	Feces	16S rRNA gene sequencing	↑ Bacteroidetes, ↓Firmicutes & *Saccharbacteria*. ↑*Parabacteroides* & *Blautia*, ↓*Prevotellaceae*, *Alistipes* & *Ruminococcaceae*
		2. FMD: fasting with the (30% of the daily calorie intake of control group) for 1 week, followed by ad libitum feeding for another week			↓ fasting blood glucose, ↓hepatic steatosis, ↓loss of pancreatic islets and β cells
Kim JN, 2019	Three ruminally cannulated Holstein steers	1.Controls: the ruminal fluids 2 h after the morning	Rumen	Denaturing gradient gel electrophoresis and quantitative polymerase chain reaction	↓ *Anaerovibrio lipolytica*, *Eubacterium ruminantium*, *Prevotella albensis*, *Prevotella ruminicola*, and *Ruminobacter amylophilus*
		2.Fasting: the ruminal fluids feeding and 24 h after fasting			
Li T, 2019	Crucian Carp fish	1. Controls: fed to satiation twice per day	Gut	16S rRNA gene amplicon sequencing	In IF groups: ↑alpha diversity and ecosystem stability of gut microbiota
		2.IF: fasted for 5 days followed by 5 days of re-feeding			↑ *Bacteroides*, *Akkermansia*, and *Erysipelotrichaceae*, ↑growth performances, immune function
		3. Long term fasting (LF): not fed anything during the whole experimental period			In LF group: ↑ Proteobacteria, *Gammaproteobacteria*, *Vibrio*, other *Vibrionaceae*
Miyamoto J, 2019	Wild-type and Gpr43 − / − mice	1.IF: alternating 24 h periods (15 cycles for 1 month) of free access to diet followed by 24 h fasting	Cecum	16S rRNA amplicon sequencing	IF group: ↑Bacteroidetes& Verrucomicrobia
		2. Eucaloric ketogenic diet: ketogenic diets for 6 weeks			↑total plasma ketone body ↓total plasma & cecal short chain fatty acids (SCFA)
Park S, 2019	Male Sprague Dawley rats: Alzheimer’s disease (AD) model	1. Controls: normal diet	Feces	16S rRNA amplicon sequencing	IF group: ↓ *Clostridales*, ↑*Lactobacillales*
		2. Ketogenic diet			Ketogenic group: ↑the relative counts of Proteobacteria especially *Enterobacteriales*
		3. High carbohydrate diet			IF and high carbohydrate groups, but not ketogenic group:
		4.IF: normal diet with intermittent fasting			↓ the hippocampal amyloidβ deposition,↑ memory function
Rangan P, 2019	8-weeks-old female C57BL/6 mice: inflammatory bowel diseases mouse model	1. Controls: standard diet	Feces	16S rRNA gene sequencing	FMD: ↑ Microbial strains associated with Tcell regulation and gut regeneration
		2. Fasting-mimicking diet (FMD): control diet plus 2 cycles of FMD			↓ intestinal inflammation, ↑stem cell number,
		3. water-only fasting (WF): control diet plus 2 cycles of WF			↑ *Bifidobacteriaceae*, ↓*Lactobacillaceae*
					WF: ↑regenerative, ↓ inflammatory markers without reversing pathology
Zhou ZH, 2019	6-week-old male C57BL/6 J mice Parkinson’s disease (PD) model	1. Controls: normal diet (12 kcal/day)	feces	16S and 18S rRNA gene sequencing	FMD: ↑ Firmicutes, Tenericutes, Opisthokonta, ↓Proteobacteria
		2. Fasting-mimicking diet (FMD) day 1: 50% of the standard daily calorie intake day 2–3: 10% of the standard daily calorie intake			↑neuroprotective effect for PD
Deng Y, 2020	3-week-old male C57BL/6 J mice:diet-induced obese model	1.controls: fed with ad libitum (AL) diet (n = 15)	feces	16S rDNA gene amplicon sequencing	↑community diversity in gut flora
		2. IF: 24-h fasting for 30 days (n = 15)			↓ Firmicutes to Bacteroidetes ratio
					↑ *Allobaculum*
					↓fat accumulation, ↑ white fat conversion to beige
Li L, 2020	7-week-old male C57BL/6JLvri mice	1. Controls: fed with ad libitum (AL) diet (n = 15)	Feces	next-generation sequencing	↑level of *Akkermansia* & ↓level of *Alistipe* in 16 h fasting group
		2. IF: divided in 3 groups of fasting: 12, 16 and 20 h (n = 15 per group)		of 16S r RNA gene amplicons	↓ cumulative food intake in the 16 and 20 h fasting groups
Liu Z, 2020	homozygous Leprdb/db mice (diabetic), heterozygous Leprdb/m, mice (non- diabetic)	1.controls: fed with ad libitum (AL) diet	Feces	16S rRNA gene v3–v4 amplicon	↑ villi length and the muscularis thickness. ↓ gut leakage, ↑cognitive function
		2. IF: deprived of food for 24 h for 28-day		Sequencing	↑ microbiome alpha diversity
					↑*Lactobacillus* and butyrate-producing *Odoribacter*. ↓*Enterococcus*, *Streptococcus*, *Candidatus Arthromitus*, *Rummeliibacillus*, *Enterococcaceae*, *Leuconostocaceae*
Ye Y, 2020	8-week-old male Kunming mice	1. High-fat diet ad libitum	Feces	16S rRNA gene V3–V4	↓ weight gain, ↓liver steatosis, ↓hepatic levels of triglycerides
		2. Time-restricted high-fat diet restricted to an 8-h temporal window per day for 8 weeks		Sequencing	↑ Bacteroidetes*,* ↓ Firmicutes
Zhang X, 2020	7–8 week –old male C57BL/6 mice: colitis mouse model	1.Controls: standard diet	Feces	16S rRNA gene	ADF, TRF & IER: ↑*Escherichia*
		2. Alternate-day fasting (ADF): 24 h feeding/fasting		sequencing	TRF and IER: ↓*Escherichia*
		3. Time restricted feeding (TRF): fed 8 h per day, fed at 24:00 p.m. and fasting at 8:00 a.m. in the morning			TRF and IER, but not ADF: ↓*Gammaproteobacteria*, *Enterobacteriaceae*, *Shigella* & *Escherichia coli,*↑ *Christensenellaceae*
		4. Intermittent energy fasting (IER): two cycles of four days of IER diet from day 11–14 and day 29–32			↑SCFAs generation-related microbes: *Rikenellaceae*, *Lactobacillus*, *Coproccus* & *Ruminococcus*
					IER but not TRF and ADF: ↓*Peptostreptococcaceae*
Shi H, 2021	5 weeks old, WKY and SHRSP hypertensive stroke-prone rats	1. Controls: ad libitum feeding 2.every other day fasting (EODF) group: alternating 24 h of ad libitum food access followed by 24 h fasting for 10 weeks	Cecum & plasma	Shotgun sequence analysis of the microbiota and untargeted metabolomics	↑Bacteriodetes & Actinobacteria, ↓proteobacteria
					↑ microbial bile acid metabolism genes: 7α-dehydroxylase and bile salt hydrolase
					↓ body weight & systolic blood pressure

**Table 2:** Summary of the human studies investigating the effects of fasting on gut microbiota alterations

First author, year	Study design	Study Subjects	Intervention	Biospecimen	Microbiota analyzing methods	Main findings	Quality assessment score
Remely M, 2015	Intervention pre-post design	13 overweight individuals	One-week Buchinger fasting program* with laxative treatment followed by a 6 week intervention with a probiotic formula	Stool	16srDNA with a quantitative real time polymerase chain reaction	No significant changes in total bacteria, or of Bacteroidetes*, Prevotella, Clostridium cluster XIVa*, or *Clostridium cluster IV.* ↑*Faecalibacterium prausnitzii, Akkermanisa* and *Bifidobacteria*	1/5
He Y, 2019	Intervention pre-post design	16 healthy individuals, Age: 18–40 years	six individuals subjected to a water-only fast and ten individuals receiving a juice fast, both for seven days	Stool	16S ribosomal RNA gene	Water-only fasting changed the bacterial community, ↑more homogenous gut microbiomes, *↓Fusobacterium*. *↓*colorectal cancer	1/5
Mesnage R, 2019	Intervention pre-post design	15 healthy men, Age: 18–70 years	10 days Buchinger fasting program with daily energy intake of about 250 kcal and an enema every 2 days	Stool	16S rRNA gene amplicon sequencing	↓ *Lachnospiraceae,Ruminococcaceae. ↑* Bacteroidetes, Proteobacteria *(Escherichia coli, Bilophila wadsworthia*)	1/5
Ozkul C, 2019–2020**	Intervention pre-post design	9 adult subjects	Ramadan fasting consisting of 17 h of fasting/day during a 29-day period	Stool	qPCR assay	↑ *A. muciniphila* and *B. fragilis ↓*Serum fasting glucose and total cholesterol levels	1/5
				Stool	16S rRNA amplicon sequencing	↑Microbial richness. *↑Butyricicoccus, Bacteroides, Faecalibacterium, Roseburia, Allobaculum, Eubacterium, Dialister, Erysipelotrichi*	1/5
Balogh A, 2020	Randomized controlled trial	Hypertensive metabolic syndrome patients 1.control (n = 36) 2.fasting (n = 35)	1. Controls: Dietary Approach to Stop Hypertension (DASH) diet. 2. Buchinger fasting protocol followed by DASH diet for 5 days	Stool	16S rRNA sequencing or shotgun sequencing	↑*Clostridial* Firmicutes* ↓butyrate producers such as F. prausnitzii, E. rectale and C. comes* at first, which were reverted after three months. ↑ *Odoribacter* species ↑ propionate production capacity, mucin degradation gene modules	3/5
Gable K, 2020	Intervention pre-post design	14 obese adults	a daily 8-h time restricted feeding (8-h feeding window/ 16-h fasting window) for 12 weeks	Stool	16S rRNA gene sequencing	Gut microbiota phylogenetic diversity remained unchanged. No significant alterations in any phyla	1/5
Lilja S, 2020	Randomized controlled single-blinded trial	154 healthy individuals 1.Buchinger fasting (n = 20) 2.fasting mimetic (n = 100) 3.control (n = 31)	1.Buchinger fasting: 250 cal a day for 5 days 2.Fasting mimetic: routine diet with supplement of prebiotic and secondary plant ingredients 3 months 3.controls: placebo	Stool	Ilumnia sequencing and mass spectrometry	Buchinger fasting group: ↑distribution of Proteobacteria*,* ↓Firmicutes/Bacteroidetes ratio Fasting mimetic supplementation group: ↑Actinobacteria	3/5
Guo Y, 2021	Randomized controlled trial	39 patients with metabolic syndrome 1.control (n = 18) 2.fasting (n = 21)	"2-day" modified fasting (69% calorie reduction compared to non-fasting days) for 8 weeks	Stool	16S rRNA gene sequencing	↓fat mass, oxidative stress, inflammatory cytokines, ↑vasodilatory parameters. Significant changes in gut microbiota communities: ↑ *Rumonococcaceae, Roseburia*, and *Clostridium* ↑production of short-chain fatty acids, ↓ lipopolysaccharides	3/5
Maifeld A, 2021	Randomized controlled trial	Hypertensive patients with metabolic syndrome 1.controls 2. fasting	1. Controls: Dietary Approach to Stop Hypertension (DASH) 2. fasting: 2 days 1200 kcal/day, 5-days 300–350 kcal/day derived from vegetables followed by a modified DASH	Stool	16S rRNA gene sequencing	↓*Bifidobacterium, ↑Bacteroides & Anaerotruncus & Alistipes* ↑Propionate production capacity, mucin degradation gene modules ↓body weight and blood pressure	3/5
Su J, 2021	Intervention pre-post design	Healthy non obese young and middle age individuals from two cohorts 1.fasting (n = 57; 27 middle age & 30 young) 2. age- & body weight–matched controls (n = 10)	Ramadan fasting consisting of 16 h of fasting/day during a 30-day period Control: routine diet/no fasting	Stool	16S rRNA gene sequencing	*↑Clostridiales*, *Lachnospiraceae* and *Ruminococcaceae* families *↑* butyric acid–producing *Lachnospiraceae* ↓*Prevotellaceae* ↓body weight, fat mass & blood glucose. ↑urea, creatinine	1/5

*Buchinger fasting program; Fat intake (g/d): 0.2, Protein intake (g/d): 1.8, Carbohydrate intake (g/d): 56.2, Fibre intake (g/d): 1.1, Energy intake (kcal/d): 234.4, Total fluid: 2–3 L per day

**Results are published in two articles

### Gut microbiota alterations during fasting in animal models

Animal studies were evaluated starvation-induced changes in the microbiome in mouse models, fruit flies (Drosophila melanogaster), fish (Crucian Carp fish), and the steer rumen or compared these alterations among different animals representing five classes of vertebrates including tilapia, toads, geckos, quail, and mice. The alterations of gut microbiota during fasting in animal models with different metabolic complications, digestive problems, and neurodegenerative diseases are presented in Table 1.

Kim JN, et al. evaluated the steer ruminal fluid by an in vitro technique and showed that 24 h fasting led to changes in the microbiota and activity in the rumen. The amount of *Anaerovibrio lipolytica*, *Eubacterium ruminantium*, *Prevotella albensis*, *Prevotella ruminicola*, and *Ruminobacter amylophilus* decreased after fasting. These changes caused higher total gas production, ammonia, and microbial protein production [18].

Two studies examined the effect of fasting on colonic microbiome of five classes of vertebrates including fishes (tilapia), amphibians (toads), reptiles (leopard geckos), birds (quail), and mammals (mice). Kohl KD, et al. in a comparative study across five classes of vertebrate hosts demonstrated that prolonged fasting [28 days] led to unique and shared alterations in microbial taxa across hosts. *Lactobacillus* and *Prevotella* decreased while *Oscillospira* increased in tilapia, quail, and mice. Several shared responses of the microbiota changes were shown across hosts. For example, all tetrapods exhibited decreases in the abundances of *Coprobacillus* and *Ruminococcus* in response to fasting [19]. McCue MD, et al. showed that prolonged fasting altered the composition of microbial communities residing in the distal intestinal tract of animals representing five classes of vertebrates. The major diversity was observed in Bacteriodetes, Firmicutes, Proteobacteria, Fusobacteria and Verrucomicrobia phyla, but no detectable change was shown in distal intestine morphology [20].

Four studies compared the effect of different fasting hours on gut microbiota and heath parameters. Sonoyama K, et al. compared 96 h fasting and hibernation conditions in Syrian Hamsters and showed that the gut microbiota responded differently to fasting and hibernation. *Akkermansia muciniphila*, a mucin degrader, increased in fasting group but not in hibernation while *Clostridia* increased in hibernating group [21]. Catterson JH, et al. showed that intermittent fasting with the pattern of 2-day fed and 5-day fasted for 40 days in fruit flies (*Drosophila melanogaster*), improved age-related pathologies and gut health due to reduced relative abundance of some harmful bacteria. However, every-other-day fasting for 30 days showed no significant effect [22]. Li T, et al. compared the effect of intermittent fasting (fasted for 5 days followed by 5 days of re-feeding) and long term fasting (not fed anything during the whole experimental period; 60 days) in Crucian Carp fish. In intermittent fasting group, an improvement was shown in alpha diversity and ecosystem stability of gut microbiota. *Bacteroides*, *Akkermansia*, and *Erysipelotrichaceae* increased and led to improvement in growth performances and immune function. However, in long term fasting group, the lowest mean of every growth parameter was observed. Moreover, in this group some operational taxonomic units (OTUs) belonging to Bacteroidetes and Proteobacteria were particularly abundant. These OTUs correlated positively with malondialdehyde, a metabolite derived from lipid peroxidation as an indicator of oxidative damages [23]. Li L, et al. evaluated the effect of daily fasting hours on shaping gut microbiota in mice. These mice have been divided in 3 groups of 12, 16 and 20 h fasting. The composition of gut microbiota was altered by all these types of fasting diets. At genus level, 16 h fasting increased the level of *Akkermansia* and decreased the level of *Alistipes*. These effects disappeared after the cessation of fasting. No taxonomic differences were identified in the other two groups [24].

Three studies have performed on diabetic mouse models. Beli E, et al. compared the diabetic mouse fed with ad libitum (AL) diet with ones fasted on an every other 24-h interval. After 7 months, mice in the fasting group showed an increase in Firmicutes and a decrease in Bacteroidetes and Verrucomicrobia phyla. Alterations in the microbiome were associated with increases in gut mucin, goblet cell number, villi length, and tauroursodeoxycholate bile acid and reductions in plasma peptidoglycan, retinal TNF-α mRNA and diabetic retinopathy [25]. Lui Z, et al. showed that intermittent fasting by depriving of food for 24-h in diabetic mice led to increase the villi length and the muscularis thickness and decrease gut leakage. Moreover, the microbiome alpha diversity and abundance of *Lactobacillus* and butyrate-producing *Odoribacter* increased while the relative abundance of *Enterococcus*, *Streptococcus*, *Candidatus arthromitus*, *Rummeliibacillus*, unknown *Enterococcaceae*, and *Leuconostocaceae* decreased. These changes resulted in improving cognitive function in diabetic mice [26]. Wei S, et al. tested a fasting mimicking diet on a genetic model of type 2 diabetic mouse. The administration of every other week fasting diet for 8 weeks led to reconstruction of gut microbiota including increased Bacteroidetes, *Parabacteroides*, *Blautia* and decreased Firmicutes, *Saccharbacteria*, *Prevotellaceae*, *Alistipes* and *Ruminococcaceae* which was correlated with lowering the fasting blood glucose, hepatic steatosis and loss of pancreatic islets and β cells [27].

Three studies have been conducted on diet-induced obese mouse model. Dengy Y, et al. indicated that 24-h fasting for 30 days increased the bacterial diversity in the intestinal flora, enriched the relative abundance of *Allobaculum*, and decreased the ratio of Firmicutes to Bacteroidetes which led to lowering fat accumulation and increasing white fat conversion to beige [28]. In contrast, Li G, et al. showed that every other day fasting regimen in diet-induced obese mouse increased the operational taxonomic unit abundance of Firmicutes, but decreased the most other phyla including Bacteriodetes and Actinobacteria. So the ratio of Firmicutes to Bacteroidetes increased which improved beiging of white adipose tissue and subsequently ameliorated metabolic disorders [29]. Ye Y, et al. showed that time-restricted high-fat diet in Kunming mice increased Bacteroidetes and decreased Firmicutes level compared to high-fat diet ad libitum. The weight gain, liver steatosis, hepatic levels of triglycerides also decreased in time-restricted high-fat diet group [30].

One study performed on hypertensive stroke-prone rats by Shi H, et al. They showed that every other day fasting (EODF) for 10 weeks had beneficial effect on body weight and systolic blood pressure through manipulation of gut microbiota. EODF increased abundance of Bacteriodetes, and Actinobacteria and expression of two major microbial bile acid metabolism genes, 7α-dehydroxylase and bile salt hydrolase, and decreased Proteobacteria abundance [31].

Two interventions conducted on inflammatory bowel disease and colitis mouse model. Rangan P, et al. showed that fasting mimicking diet increased the microbial strains known to be associated with T cell regulation and gut regeneration and improved intestinal inflammation, stem cell number, and frequency of the protective gut bacteria including *Bifidobacteriaceae*. The water only fasting also could decrease inflammatory markers without reversing pathology. They indicated that the major microbiota changes require both the fasting mimicking diet and several days of re-feeding with a normal diet [32]. In another study, Zhang X, et al. compared three kinds of fasting in colitis mouse model: 1. alternate-day fasting (ADF): 24-h feeding/fasting circle, 2. time restricted feeding (TRF): fed for only 8 h per day, and 3. intermittent energy fasting (IER): two cycles of four days of IER diet. ADF, TRF and IER, all affected the gut microbiota composition. At the genus level, *Escherichia* increased in colitis mice compared with the controls. However, TRF and IER treatment significantly suppressed the levels of *Escherichia* in colitis mice. TRF and IER, but not ADF decreased *Gammaproteobacteria*, *Enterobacteriaceae*, *Shigella*, and *Escherichia coli* and increased *Christensenellaceae*. Besides, IER but not TRF and ADF decreased *Peptostreptococcaceae*. The enrichment of SCFAs-generating microbes including *Rikenellaceae*, *Lactobacillus*, *Coproccus*, and *Ruminococcus* were partly improved by the TRF and IER but not ADF in colitis mice gut [33].

Three studies performed on neurodegenerative diseases in mouse model. Cignarella F, et al. showed that every other day fasting diet in multiple sclerosis (MS) mice, increased the level of *Lactobacillaceae*, *Bacterioidaceae* and *Prevotellaceae* families and ameliorated the clinical course and pathology of the MS and decreased inflammation, demyelination and axonal damage [34]. Park S, et al. compared every other day fasting diet with ketogenic and high carbohydrate diet in Alzheimer’s disease (AD) mice. Fasting diet decreased *Clostridales* and increased *Lactobacillales*. Ketogenic diet exacerbated gut dysbiosis by increasing Proteobacteria, but carbohydrate diet improved it by elevating Bacteriodetes. In fasting and carbohydrate group, but not ketogenic group, memory function, insulin resistance, neuroinflammation and gut microbiota improved [35]. Zhou ZH, et al. showed that fasting-mimicking diet in Parkinson’s disease (PD) mice modulated the shifts in gut microbiota composition. The abundance of Firmicutes, Tenericutes, Opisthokonta increased but Proteobacteria decreased due to fasting diet. These changes had neuroprotective effect in PD mice [36].

As a whole, the results of animal studies indicate the positive effect of fasting on metabolic disorders including obesity, diabetes, hypertension, as well as inflammatory and neurodegenerative diseases through alteration of gut microbiota. Moreover, the various types and durations of fasting regimens were shown to have different effects on gut microbiota and health parameters, indicating the needs for further study.

### Gut microbiota alterations during fasting in human studies

**Fig. 2:**
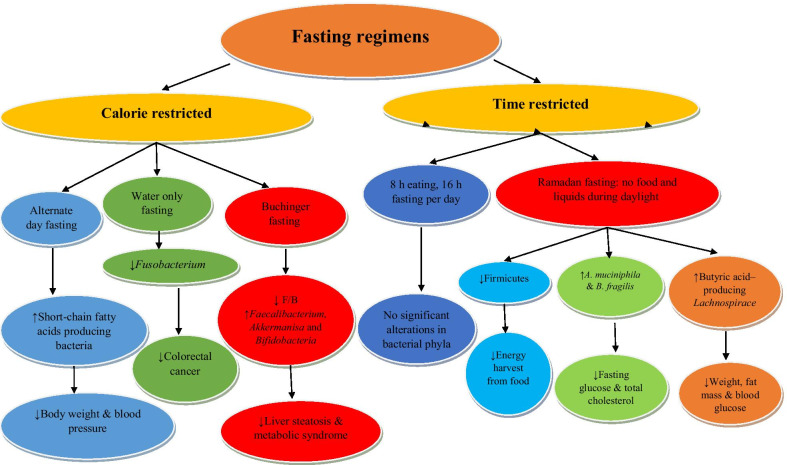
Effect of different types of fasting regimen on the gut microbiota composition and health parameters

Totally 11 studies dealing with fasting and the gut microbiota in humans were included in this systematic review (Table 2). The fasting diets differed in structure and duration in different studies (Fig. 2). Four studies examined Buchinger fasting program. This program involve a daily energy intake of about 1046 kJ (250 kcal) from fat 0.2 g/d, protein 1.8 g/d, carbohydrate 56.2 g/d, fiber 1.1 g/d and total fluid 2–3 L per day [37]. Remely M, et al. analyzed the effect of one-week Buchinger fasting program with laxative treatment followed by a 6 week intervention with a probiotic formula in overweight individuals. No significant changes in abundance of total bacteria, or Bacteroidetes, *Prevotella*, *Clostridium* cluster XIVa, or *Clostridium* cluster IV were found, although *Faecalibacterium prausnitzii*, *Akkermanisa* and *Bifidobacteria* increased in abundance over the study period. No significant improvements of eating habits were reported, although physical activity improved due to the intervention [38]. Mesnage M, et al. showed that 10 d of Buchinger fasting led to alterations in human gut microbiota composition. The abundance of bacteria known to degrade dietary polysaccharides such as *Lachnospiraceae* and *Ruminococcaceae* decreased while the ones that use derived energy substrates including Bacteroidetes and Proteobacteria increased. These changes were associated with host energy metabolism [39]. Lilja S, et al. compared 5 days periodic Buchinger fasting intervention with 3 months shot supplementation, a drink formula, containing secondary plant ingredients considered to activate sirtuins. In the Buchinger fasting group the frequency of Proteobacteria increased while the Firmicutes/Bacteroidetes ratio decreased. In the supplementation group, Actinobacteria, a butyrate producing bacterial phylum, increased. Both interventions revealed results with beneficial outcomes for human health and confirm the effects of fasting on longevity associated mechanisms [40]. Balogh K, et al. revealed that in hypertensive metabolic syndrome patients, a 5-day fast according to Buchinger protocol improved gut microbiome, and immune homeostasis with a sustained beneficial effect on body weight and blood pressure. In this study, at first many Clostridial Firmicutes increased while butyrate producers such as *F. prausnitzii*, *E. rectale* and *C. comes* decreased which had also reverted after three months. Functional analysis of gut microbiota showed enhancement of propionate production and mucin degradation capacities as a result of Buchinger fasting [41].

He Y, et al. compared two models of fasting, water-only fast and juice fast, both for seven days. It has been shown that water-only fasting increased more homogenous gut microbiome and led to lower *Fusobacterium* which promotes colorectal cancer. These changes remained in 5 out of 6 subjects even after returning to their normal diet [42].

Gabel K, et al. examined the effects of intermittent fasting on changes in the gut microbiome in obese adults. The participants received a daily 8-h time restricted feeding intervention (8-h feeding window/16-h fasting window) for 12 weeks. Fecal microbiota analysis showed no significant difference in gut microbiota phylogenetic. Although body weight decreased, no significant alterations in the abundance of Firmicutes, Bacteroidetes, or any other phyla were detected after 12 weeks [43]. Guo Y, et al. in a randomized clinical trial evaluated the effects of intermittent fasting on cardiometabolic risk factors and the gut microbiota in patients with metabolic syndrome. Patients divided into intervention and control groups. Controls received their normal diet and intervention consisted of 8 weeks of "2-day" modified intermittent fasting. On fasting days, participants reduced 69% of their calorie intake compared to nonfasting days. Intermittent fasting induced significant changes in gut microbiota. The production of short-chain fatty acids increased and the circulating levels of lipopolysaccharides decreased. These alterations were significantly associated with improved cardiovascular risk factors and reduced fat mass, ameliorated oxidative stress, modulated inflammatory cytokines and improved vasodilatory parameters and led to distinct genetic shifts of carbohydrate metabolism in the gut community [44]. Maifeld A, et al. examined a fasting program which started with two calorie-restricted vegan days (max 1200 kcal/day), then 5-days with a daily nutritional energy intake of 300–350 kcal/day, followed by a modified DASH diet in patients with metabolic syndrome. Fasting altered the gut microbiome, impacting bacterial taxa and gene modules associated with short-chain fatty acid production. These changes caused a sustained beneficial effect on body weight and blood pressure [45].

Three other studies assessed the effect of Islamic or Ramadan fasting on the gut microbiome. Islamic fasting is a kind of intermittent fasting regimen, which includes a fasting period from sunrise to sunset during the holy month of Ramadan. Oskul C, et al. compared the stool samples of adult subjects before (baseline) and at the end of the Ramadan month consisting of 17 h of fasting/day during a 29-day period. After Ramadan fasting, an increase in microbial richness was observed and the frequency of *Butyricicoccus*, *Bacteroides*, *Faecalibacterium*, *Roseburia*, *Allobaculum*, *Eubacterium*, *Dialister*, and *Erysipelotrichi* increased. The bacterial species most affected by the Ramadan fasting was *Butyricicoccus pullicaecorum*, *Akkermansia muciniphila* and *Bacteroides fragilis* which led to reduction of serum fasting glucose and total cholesterol levels [15, 46]. Su J, et al. evaluated the effect of Ramadan fasting (16 h of fasting/day during a 30-day period) on the gut microbiota. The study subjects were young (average age: 19y) and middle aged (average age: 40y) healthy non-obese individuals. Ramadan fasting was led to the substantial remodeling of the gut microbiota. The *Clostridiales*, *Lachnospiraceae, Ruminococcaceae* families and butyric acid–producing *Lachnospiraceae* increased while *Prevotellaceae* decreased. These alterations accompanied by changes in various physiologic parameters and reduced energy intake. Increment in *Lachnospiraceae* abundance provided a positive effect on physiologic surrogate markers such as blood glucose and body weight and body fat mass. These effects disappeared when fasting was stopped but the abundance of some species of *Escherichia coli* and *Ruminococcaceae* family (*Faecalibacterium*, unidentified *Enterobacteriaceae*, and unidentified *Ruminococcaceae*) was still higher even after the cessation of the fasting [47].

## Discussion

In this systematic review, we provided an overview of the existing literature regarding the gut microbiota during fasting. Most studies have reported the positive effects of fasting diets on modulating intestinal microbiota composition, improving host functions and slowing disease progression. Current evidence suggests that fasting might be a successful intervention to prevent and manage metabolic disorders with greater reduction in anthropometric parameters, glycemic indices and lipid profile compared with standard continuous energy restriction diets [48–50]. Fasting diets also decrease inflammation, which are believed to protect against metabolic, neurodegenerative and age related diseases [51, 52]. It was demonstrated in animal models that fasting diets can markedly reduce systemic inflammation through decreasing mRNA expression levels of inflammatory cytokines and chemokines in white adipose tissue including IL-6, IL-1Ra, IL-2,MCP-1, and CXCL16 [53]. Regarding the role of microbiome in modulating adiposity and protecting against the development of obesity-related metabolic dysfunction, recently the factors affecting the regulation of gut microbita have received much attention. Preliminary animal models suggest that intermittent fasting may be one of these modulators [54]. Intermittent fasting in mice induced white adipose tissue (WAT) beiging, weight loss, and changes in the gut microbiota, including a decrease in the Firmicutes to Bacteroidetes ratio [13]. This was associated with improvements in liver steatosis and metabolic syndrome components. However, in microbiota-depleted mice, fasting did not improve obesity or liver steatosis, thus suggesting that the gut microbiota alteration plays a key role as an underlying mechanism in fasting-induced health benefits [55]. Upon fasting, several beneficial bacteria including *Lactobacillus* and *Bifidobacterium* shifted significantly in abundance. *Lactobacillus* and *Bifidobacterium* are documented as useful strains for human health in lots of clinical trials on the adult population [56]. Some species such as *Escherichia coli* that associated with endothelial dysfunction and metabolic syndrome depleted, however, some taxa including *Odoribacter* which negatively associated with both blood pressure and vascular stiffness bloomed during fasting. These findings suggest that fasting could promote health through the gut microbiome [41, 57, 58]. Although the complexity of the relationship between fasting and gut microbiota is difficult to interpret, however there are a number of host-driven mechanisms may justify this association. Fasting can influence microbial community by preventing the production of antimicrobial proteins and other aspects of mucosal immune function in the host [59, 60]. Moreover, fasted animals tend to exhibit higher gut pH compared with fed animals which in turn have effects on microbial growth [61]. Fasting also results in alteration of mucus production which can alter microbial diversity, because several gut microorganisms get rich on mucus and differential production of glycans may support the growth of different types of microorganisms [62, 63]. Another mechanism is the reduction of the intestine size in many fasting animals. Size reduction causes a ‘housing crisis’ for microorganisms that could result in increased competition for space [64]. Over the past decades, human and animal studies have shown that timing of meal intake is as important as the composition of the diet and caloric quantity to prevent obesity and its complications [10, 11]. Studies reveal that changes in daily feeding and fasting rhythms can alter the gut microbiota in animal models [65]. There is a multifaceted relationship between microbiota and meal timing: first, intestinal epithelia cells’ internal circadian clock influences daily glucocorticoid production under the control of the pituitary-adrenal axis, and this rhythm is influenced by microbiota status; second, an alteration of microbiota could lead to a disrupted corticosteroid circadian rhythm influencing food uptake. Consuming food outside the normal feeding phase may disturb normal peripheral and central clocks resulted in increased risks of metabolic and cardiovascular diseases [66, 67]. So the effect of fasting and feeding patterns on metabolism can be closely associated with alterations in gut microbiota [14, 68].

Nowadays several modifications of fasting diets have gained popularity as they offer impressive health benefits. However, it is unlikely that all fasting diets lead to the same physiological changes because of their different fasting and feeding patterns. A number of recent studies have suggested that fasting diets are effective, as is traditional calorie restriction for weight loss and improving health parameters [69]. However, it is still unclear whether fasting improves gut microbiota and health indicators in the same ways as calorie restriction. This review provides an overview of the effects of different kinds of long or intermittent fasting on the abundance of different gut bacteria. There are various types of fasting programs that restricted meal time or calorie to improve body composition and overall health. One of the common calorie-restricted fasting is the Buchinger program which involves a daily energy intake of about 250 kcal [37]. The other examples of such amodification are alternating eating a day and then fasting the next day or 2 days fasting per week. During the fasting period, one can completely eliminate foods or reduce calorie intake to a minimum [70]. In time-restricted fasting, people abstain from eating for a specific period of time and then eat meals in the feeding window. The periods of fasting or eating windows are various. The most common modification is eating for 8 h, followed by fasting for the next 16 h. Religious fasting is another example, which is a wide range of fasts undertaken for religious or spiritual purposes.

Regarding Buchinger fasting, results of studies showed increase of Proteobacteria abundance and decrease of Firmicutes to Bacteroidetes ratio at phylum level. Besides, *Faecalibacterium prausnitzii, Akkermanisa* and *Bifidobacteria* has been reported to increase while results about the butyrate-producing bacteria were contradictory [38–41]. It has been shown that enhancement of *Odoribacter* abundance following the Buchinger regimen was negatively associated with systolic blood pressure and vascular stiffness [41]. A novel class of sphingolipid compounds, namely sulfonolipids with potential anti-inflammatory effects was identified as a bacterial metabolite which released from *odoribacter* [71]. It seems that more investigation is needed to determine the functional roles of these bacterial lipids on metabolic health effects of fasting. Moreover, alternate day fasting was associated with metabolic health through enhancing the capacity of short-chain fatty acids production of gut microbiota as well as decreasing lipopolysaccharides release and ameliorating inflammatory status as a consequence [44].

Few research has been done on the gut microbiota alteration following 8-h feeding/16-h fasting as the most common time-restricted fasting regimen up to now [43]. Therefore, the possible mediating effect of gut microbiota in fasting diet-induced metabolic health should be assessed in further clinical trial. In Ramadan, millions of Muslims undertake one month of fasting in observance of this religious obligation and abstain from food and liquids during daylight hours from dawn to sunset [72]. Several health benefits have been attributed to Ramadan fasting as a prevalent type of intermittent fasting. Studies reveal that Ramadan fasting elicits a significant decline in body weight and fat mass which in turn leads to better control of metabolic disorders including diabetes, hypertension, hyperlipidemia and etc. [73–77]. Besides to the reduction in meal frequency, there is a metabolic shift toward the main use of fatty acids as fuel for synthesizing adenosine triphosphate (ATP) during Ramadan fasting causing body fat reduction, improving functional capacity, resting energy expenditure and blood glucose homeostasis [78]. The optimization of energy reserves, decreased secretion of anabolic hormones and increased secretion of catabolic hormones such as adrenaline and glucagon have been proposed as underlying mechanisms for beneficial effect of Ramadan fasting on metabolism [79]. Moreover, Ramadan fasting had considerable effects on the gut microbiota composition. According to the results of studies, *A. muciniphila, B. fragilis, Bacteroides* and butyric acid–producing *Lachnospiraceae* which have been largely accepted as the major members of the healthy gut microbiome increased after Ramadan fasting [46, 47]. The relative abundance of *A. muciniphila* as a mucin-degrading bacterium which resides in the mucus layer is inversely correlated with body weight [80, 81]. Human studies have demonstrated an increased abundance of *A. muciniphila* after calorie restriction in obese patients along with the healthier metabolic outcomes [38, 82]. *A. muciniphila* strongly adheres to the mucosal layer, so it may remain relatively stable during the dietary modifications and subsequent changes in intestinal passage/flow rates and alteration of defecation regimens [83]. Regarding *B. fragilis,* its increase in overweight adolescents after reduction in energy intake has been reported [85]. *Bacteroides* genus, as an important member of healthy gut microbiota, could increase the tolerance to changes in the intestinal tract and has high capability to decrease oxygen levels in the gut lumen and high potency to metabolize complex polysaccharides and fatty acids. This genus has the unique ability to switch their transcriptional profile to use host-derived glycans in the absence of polysaccharides and glycoproteins, such as the fasting periods [85]. The property of rapid adaptation to nutrient availability and high survival of *Bacteroides* may contribute to its resistance to time-restricted energy intake and may lead to increased dominance after the depletion of the other bacterial groups during fasting. Furthermore, Ramadan fasting provides an obvious possible mechanistic explanation for health effects associated with intermittent fasting via upregulating the butyric acid–producing *Lachnospiraceae* [47]. Evidence presented that butyrate has immunomodulatory properties and could regulate energy homeostasis[86]. After Ramadan fasting, a decreasing trend was observed in Firmicutes and *Enterobacteriaceae* abundance. Firmicutes is responsible for increased energy harvest from foods and mostly is associated with obesity despite its anti-inflammatory and butyrate source features [87]. Besides, *Enterobacteriaceae* is also known as a source of endotoxin production, and its abundance is closely related with decreased gut permeability [88].

This study comprehensively reviewed existing animal and human surveys regarding the effect of different types of fasting diets on gut microbiota alterations. However, it has some limitations; the human studies consisted of generally small size and they were disparate in their purposes, methodologies and studied population in terms of diet, weight, geographic location, and host genetics which can affect the pattern of the gut microbiome. There are substantial differences in gut microbiota composition between various ethnic groups that were only partly explained by sociodemographic status, lifestyle, and dietary patterns. Therefore, ethnic differences should be taken into account when studying associations between fasting or other dietary regimens and the gut microbiota composition. Moreover, many of the studies failed to account for common confounders, such as the effects of smoking, and physical activity. Furthermore, in the case of Islamic fasting, the timing of Ramadan moves throughout the year, in accordance with the phases of the moon which can have different effects on gut microbiota composition and health parameters.

Regarding the quality of included clinical trials, only 4 studies had good quality based on Jadad score. However, it should be noted that due to the nature of this intervention, it is too hard or sometimes impossible that participants or staff be blinded to allocation. Moreover, most of the studies were pilot and they included no control groups. So, further well-designed randomized controlled trials are needed to have a conclusion about the effects of various fasting regimens on microbiota composition and overall well-being.

It was shown that both time and calorie restriction fasting regimens can be able to alter the taxonomic composition of gut microbiota. However, due to the using various animal models and different biospecimens regarding microbiota composition in animal studies, interpretation of the results should be done with caution. Further studies considering these issues are warranted for evaluating the exact effect of each type of fasting diets on microbiota composition of different body sites.

## Conclusion

This systematic review revealed that maintaining a correct eating time and increasing the fasting period could positively affect the gut microbiome, reduce the gut permeability and improve health. This insight was gained by considering different fasting regimens including alternate-day fasting, calorie restriction fasting programs such as Buchinger fasting, and religious fasting including Islamic or Ramadan fasting. The evidence supported the association of Ramadan fasting with improving health parameters and slowing disease progression through positive changes in gut microbiota including upregulation of *A. muciniphila, B. fragilis, Bacteroides* and butyric acid–producing *Lachnospiraceae*. However, further studies are needed to explore properly the connection between microbiota and meal frequency and timing. Although the evidence supports the hypothesis that fasting diets could promote health through the modulation of gut microbiome and totally fasting regimens could be suggested in clinical settings, the effects should be confirmed by well-designed randomized controlled trials on various target groups with different baseline characteristics and comorbidities.

## Data Availability

Not applicable.

## Abbreviations

OTUs: Operational taxonomic units
AL: Ad libitum
EODF: Every other day fasting
ADF: Alternate-day fasting
TRF: Time restricted feeding
IER: Intermittent energy restricted
MS: Multiple sclerosis
SCFAs: Short-chain fatty acids
AD: Alzheimer’s disease
PD: Parkinson’s disease
DASH: Dietary approach to stop hypertension
WAT: White adipose tissue
ATP: Adenosine tri-phosphate
